# Supplementary material for: COZOID: contact zone identifier for visual analysis of protein-protein interactions
Source: BMC Bioinformatics. 2018 Apr 6;19:125. doi: 10.1186/s12859-018-2113-6 (PMC5889581; doi:10.1186/s12859-018-2113-6)
Supplement: Supplementary file 2 — Software build. Executable binary file of the software tool. (ZIP 118,784 kb) [file 12859_2018_2113_MOESM2_ESM.zip › cozoid/java/docs/beansbinding-1.2.1-doc.zip!/org/jdesktop/el/impl/parser/SimpleCharStream.html]

SimpleCharStream (Beans Binding)


---


|  |  |  |
| --- | --- | --- |
| **Overview** \| **Package** \| **Class** \| **Use** \| **Tree** \| **Deprecated** \| **Index** \| **Help** | |  |
| **PREV CLASS**   **NEXT CLASS** | **FRAMES**    **NO FRAMES**     **All Classes** |  |
| SUMMARY: NESTED \| FIELD \| CONSTR \| METHOD | DETAIL: FIELD \| CONSTR \| METHOD |  |


---


## org.jdesktop.el.impl.parser Class SimpleCharStream

```
java.lang.Object
  org.jdesktop.el.impl.parser.SimpleCharStream
```

---

``` public class SimpleCharStream extends java.lang.Object ```

An implementation of interface CharStream, where the stream is assumed to
contain only ASCII characters (without unicode processing).

---

| **Field Summary** | |
| --- | --- |
| `protected  int[]` | `bufcolumn` |
| `protected  char[]` | `buffer` |
| `protected  int[]` | `bufline` |
| `int` | `bufpos` |
| `protected  int` | `column` |
| `protected  int` | `inBuf` |
| `protected  java.io.Reader` | `inputStream` |
| `protected  int` | `line` |
| `protected  int` | `maxNextCharInd` |
| `protected  boolean` | `prevCharIsCR` |
| `protected  boolean` | `prevCharIsLF` |
| `static boolean` | `staticFlag` |


**Constructor Summary**

`SimpleCharStream(java.io.InputStream dstream)`

`SimpleCharStream(java.io.InputStream dstream,
int startline,
int startcolumn)`

`SimpleCharStream(java.io.InputStream dstream,
int startline,
int startcolumn,
int buffersize)`

`SimpleCharStream(java.io.Reader dstream)`

`SimpleCharStream(java.io.Reader dstream,
int startline,
int startcolumn)`

`SimpleCharStream(java.io.Reader dstream,
int startline,
int startcolumn,
int buffersize)`


| **Method Summary** | |
| --- | --- |
| `void` | `adjustBeginLineColumn(int newLine, int newCol)`             Method to adjust line and column numbers for the start of a token. |
| `void` | `backup(int amount)` |
| `char` | `BeginToken()` |
| `void` | `Done()` |
| `protected  void` | `ExpandBuff(boolean wrapAround)` |
| `protected  void` | `FillBuff()` |
| `int` | `getBeginColumn()` |
| `int` | `getBeginLine()` |
| `int` | `getColumn()`             **Deprecated.** |
| `int` | `getEndColumn()` |
| `int` | `getEndLine()` |
| `java.lang.String` | `GetImage()` |
| `int` | `getLine()`             **Deprecated.** |
| `char[]` | `GetSuffix(int len)` |
| `char` | `readChar()` |
| `void` | `ReInit(java.io.InputStream dstream)` |
| `void` | `ReInit(java.io.InputStream dstream, int startline, int startcolumn)` |
| `void` | `ReInit(java.io.InputStream dstream, int startline, int startcolumn, int buffersize)` |
| `void` | `ReInit(java.io.Reader dstream)` |
| `void` | `ReInit(java.io.Reader dstream, int startline, int startcolumn)` |
| `void` | `ReInit(java.io.Reader dstream, int startline, int startcolumn, int buffersize)` |
| `protected  void` | `UpdateLineColumn(char c)` |

**Methods inherited from class java.lang.Object**

`clone, equals, finalize, getClass, hashCode, notify, notifyAll, toString, wait, wait, wait`

**Field Detail**

### staticFlag

```
public static final boolean staticFlag
```

**See Also:**: Constant Field Values

---


### bufpos

```
public int bufpos
```

---


### bufline

```
protected int[] bufline
```

---


### bufcolumn

```
protected int[] bufcolumn
```

---


### column

```
protected int column
```

---


### line

```
protected int line
```

---


### prevCharIsCR

```
protected boolean prevCharIsCR
```

---


### prevCharIsLF

```
protected boolean prevCharIsLF
```

---


### inputStream

```
protected java.io.Reader inputStream
```

---


### buffer

```
protected char[] buffer
```

---


### maxNextCharInd

```
protected int maxNextCharInd
```

---


### inBuf

```
protected int inBuf
```


**Constructor Detail**

### SimpleCharStream

```
public SimpleCharStream(java.io.Reader dstream,
                        int startline,
                        int startcolumn,
                        int buffersize)
```

---


### SimpleCharStream

```
public SimpleCharStream(java.io.Reader dstream,
                        int startline,
                        int startcolumn)
```

---


### SimpleCharStream

```
public SimpleCharStream(java.io.Reader dstream)
```

---


### SimpleCharStream

```
public SimpleCharStream(java.io.InputStream dstream,
                        int startline,
                        int startcolumn,
                        int buffersize)
```

---


### SimpleCharStream

```
public SimpleCharStream(java.io.InputStream dstream,
                        int startline,
                        int startcolumn)
```

---


### SimpleCharStream

```
public SimpleCharStream(java.io.InputStream dstream)
```


**Method Detail**

### ExpandBuff

```
protected void ExpandBuff(boolean wrapAround)
```

---


### FillBuff

```
protected void FillBuff()
                 throws java.io.IOException
```

:   **Throws:**: `java.io.IOException`

---


### BeginToken

```
public char BeginToken()
                throws java.io.IOException
```

:   **Throws:**: `java.io.IOException`

---


### UpdateLineColumn

```
protected void UpdateLineColumn(char c)
```

---


### readChar

```
public char readChar()
              throws java.io.IOException
```

:   **Throws:**: `java.io.IOException`

---


### getColumn

```
public int getColumn()
```

:   **Deprecated.** 

    :   **See Also:**: `getEndColumn()`

---


### getLine

```
public int getLine()
```

:   **Deprecated.** 

    :   **See Also:**: `getEndLine()`

---


### getEndColumn

```
public int getEndColumn()
```

---


### getEndLine

```
public int getEndLine()
```

---


### getBeginColumn

```
public int getBeginColumn()
```

---


### getBeginLine

```
public int getBeginLine()
```

---


### backup

```
public void backup(int amount)
```

---


### ReInit

```
public void ReInit(java.io.Reader dstream,
                   int startline,
                   int startcolumn,
                   int buffersize)
```

---


### ReInit

```
public void ReInit(java.io.Reader dstream,
                   int startline,
                   int startcolumn)
```

---


### ReInit

```
public void ReInit(java.io.Reader dstream)
```

---


### ReInit

```
public void ReInit(java.io.InputStream dstream,
                   int startline,
                   int startcolumn,
                   int buffersize)
```

---


### ReInit

```
public void ReInit(java.io.InputStream dstream)
```

---


### ReInit

```
public void ReInit(java.io.InputStream dstream,
                   int startline,
                   int startcolumn)
```

---


### GetImage

```
public java.lang.String GetImage()
```

---


### GetSuffix

```
public char[] GetSuffix(int len)
```

---


### Done

```
public void Done()
```

---


### adjustBeginLineColumn

```
public void adjustBeginLineColumn(int newLine,
                                  int newCol)
```

:   Method to adjust line and column numbers for the start of a token.


---


|  |  |  |
| --- | --- | --- |
| **Overview** \| **Package** \| **Class** \| **Use** \| **Tree** \| **Deprecated** \| **Index** \| **Help** | |  |
| **PREV CLASS**   **NEXT CLASS** | **FRAMES**    **NO FRAMES**     **All Classes** |  |
| SUMMARY: NESTED \| FIELD \| CONSTR \| METHOD | DETAIL: FIELD \| CONSTR \| METHOD |  |


---
